# Safranal: From an Aromatic Natural Product to a Rewarding Pharmacological Agent

**Published:** 2013-01

**Authors:** Ramin Rezaee, Hossein Hosseinzadeh

**Affiliations:** 1 Pharmaceutical Research Center, Department of Pharmacodynamy and Toxicology, School of Pharmacy, Mashhad University of Medical Sciences, Mashhad, I.R. Iran.; 2 Pharmaceutical Research Center, Department of Pharmacodynamy and Toxicology, School of Pharmacy, Mashhad University of Medical Sciences, Mashhad, Iran

**Keywords:** Crocus sativus, Review, Safranal, Saffron

## Abstract

Safranal, the main component of *Crocus sativus* essential oil, is thought to be the main cause of saffron unique odor. It is now about eighty years that this compound has been discovered and since then different scientific experiments have been done investigating its biological-pharmacological activities. Safranal effects in CNS have been more attractive to scientists and an escalating number of papers have been published regarding its neuropsychological effects. These promising properties of safranal propose its presence as a therapeutic agent in future, although there is a great need for further clinical trials and toxicological studies. In this review article, according to Scopus ®, Thomson Reuters Web of Knowledge®, Scientific Information Database (SID) ® and Pubmed ® all papers published until July 2012 were thoroughly discussed and a brief note of each study was prepared.

## Introduction

*C. sativus, *a member of Iridaceae family, commonly known as saffron, has been widely used as an aphrodisiac, antispasmodic and expectorant in the folk medicine (1). The pharmacological effects of aqueous or alcoholic extracts of *C. sativus* stigmas have been described in the literature and comprise a wide spectrum of activities, including anticonvulsant (2), antidepressant (3), antinociceptive and anti-inflammatory (4), antioxidant (5), acetylcholinesterase inhibiting (6), antitussive (7), reducing withdrawal syndrome (8), improving male erectile dysfunction (9), enhancing spatial cognitive abilities after chronic cerebral hypoperfusion (10), hypotensive (11) and antisolar (12) properties. According to chemical analysis, more than 150 chemicals are present in saffron stigmas (13) among which, all these pharmacological effects have been related to saffron main chemical compounds such as crocin, picrocrocin and safranal which are responsible for saffron exclusive color, taste and odor, respectively (14).

In this review article, physicochemical properties and pharmacological-toxicological activity of safranal is discussed thoroughly based on the literature searching (July 2012) which has been done using Scopus®, Thomson Reuters Web of Knowledge®, Scientific Information Database (SID) ® and Pubmed Database, looking for the term ‘Safranal’ in title, abstract and keywords.

***History***

**Figure 1:**
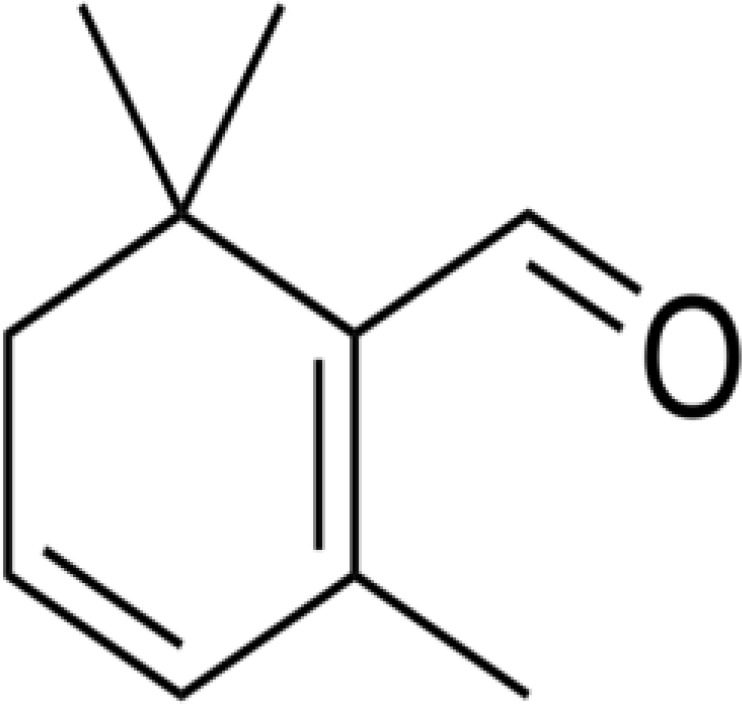
Safranal chemical structure

Beside its exclusive color, saffron presents a particular taste which is originates from its picrocrocin content. Safranal (2, 6, 6-trimethyl-1, 3-cyclohexadien-1-carboxaldehyde) or C_10_H_14_O (Figure 1) is a cyclical terpenic aldehyde produced from picrocrocin. Picrocrocin (C_6_H_26_O_7_), discovered by Kajser (1884), cracks down following acids and alkali conditions, resulting in a molecule of water and an aglycon which in turn, loses a water molecule and finally turns to safranal (15). Kuhn and Winterstein (1933), who obtained safranal through picrocrocin hydrolyzation for the first time, named this chemical as “Safranal”. 

***Physicochemical properties***

According to Merckindex (16), some physico-chemical properties of safranal are summarized in Table 1. Safranal as the most abundant chemical in saffron essential oil accounts for 60-70% of volatile fraction (17, 18). Safranal is the main compound affecting saffron characteristic aroma (19). This aroma is not found over recently picked stigmas and is produced via enzymatic and thermal degradation during the storage phase. Himeno and Sano (20) have shown that higher temperatures (80^◦^C) and less processing time (30 min) yield to a greater amount of safranal. Saffron of good quality presents 2.5% volatile compounds such as safranal (21). 

Safranal as the most abundant chemical in saffron essential oil accounts for 60-70% of volatile fraction (17, 18). Safranal is the main compound affecting saffron characteristic aroma (19). This aroma is not found over recently picked stigmas and is produced via enzymatic and thermal degradation during the storage phase. Himeno and Sano (20) have shown that higher temperatures (80◦C) and less processing time (30 min) yield to a greater amount of safranal. Saffron of good quality presents 2.5% volatile compounds such as safranal (21). 

***Natural sources of safranal***

It has been shown that geographical origin makes a great difference in safranal content of saffron (22). In Table 2, some recent research projects in which safranal has been characterized, are summarized. 

***Safranal extraction and detection***

In order to evaluate safranal content of saffron, ISO 3632 (2003) (33) proposes a spectrophotometric method in which, saffron aqueous extract absorbance is measured at 330 nm. Since safranal is not very soluble in water, the spectrophotometric method is not a very proper way of evaluation due to the interference caused by other compounds (17, 34-35). Different methods are available to extract naturally occurring volatile compounds (Table 3). It should be mentioned that greater hydrophobic properties of the extracting solvent results in better safranal extraction (36). 

**Table 1:** Safranal physicochemical properties (16)

Molecular weight	d_4_^19^	bp_1.0_	*n*_D_^19^	Soluble in:
150.22	0.9734	70^0^C	1.5281	Methanol, ethanol, petroleum ether, glacial acetic acid

**Table 2:** Plants from which safranal has been extracted

Plant	Part
*Crocus sativus *(23)	entire plant
*Centaurea sibthorpii *(24)	aerial parts
*Centaurea amanicola *(25)	aerial parts
*Centaurea consanguinea *(25)	aerial parts
*Erodium cicutarium *(26)	entire plant
*Chinese green tea *(27)	green tea trade marks
*Calycopteris floribunda *(28)	leaves
*Crocus heuffelianus *(29)	shoot primordia
*Sambucus nigra *(30)	flowers
*Lemon (* *Citrus limon) *(31)	fruits
*Achillea distans *(32)	root

***Safranal pharmacological effects***

Being famous as a flavoring and odorant chemical (40), safranal is also known as a great antioxidant (41), a protective agent against indomethacin-induced gastric ulcers (42), a natural product for protection against PTZ-induced status epilepticus (43), a pharmacological tool that is able to inhibit the acquisition and expression of morphine-induced place preference (CPP) (44) and a cytotoxic substance which is effective against specific cancer cells, *in vitro* (45). 

***Effects on central nervous system***

The aqueous or ethanolic extracts of *C. sativus* stigmas and Safranal effects on CNS have been widely studied and various benefits have been elicited i.e. anticonvulsant (2), antidepressant (3), reducing withdrawal syndrome (8), and enhancing spatial cognitive abilities after chronic cerebral hypoperfusion (10).

**Table 3:** Conventional methods for extracting volatile compounds

Extraction method
Microsimultaneous Hydrodistillation–Extraction (37)
Vacuum Headspace (18)
Supercritical Fluid Extraction (36)
Thermal Desorption (17)
Liquid Extraction With Organic Solvents (35, 38)
Ultrasound-Assisted Extraction (22, 37, 39)

*Antianxiety and hypnotic effects*

In order to evaluate safranal effects on sleep- wake cycle, pentobarbital 20 mg/kg was administered to mice and sleep-wake cycle changes and immunohistochemical expression of c-Fos in the brain were assessed following the administration of safranal (90,180 or 360 mg/kg, intragastrically). According to results, the duration of non-rapid eye movement (NREM) and the delta power activity of NREM sleep were increased and the NREM sleep latency was decreased. c-Fos expression was improved in the ventrolateral preoptic nucleus (VLPO) and decreased in the arousal histaminergic tuberomammillary nuclei (TMN). The hypnotic property of safranal was linked to potentiation of the sleep-promoting neurons in the VLPO alongside the inhibition of the wakefulness-promoting neurons in the TMN (46).

Based on the evidences from traditional use of saffron in the treatment of insomnia and anxiety, Hosseinzadeh and Noraei studied the effect of saffron aqueous extract (56, 80, 320 and 560 mg/kg), crocin (50, 200 and 600 mg/kg) and safranal (0.05, 0.15 and 0.35 ml/kg) in mice, using the Rotarod, Elevated plus maze, pentobarbital sleeping time and Open field tests. Saffron (56 and 80 mg/kg) and safranal (0.15 and 0.35 ml/kg) increased the time spent in open arms of the maze, which is the marker for anti anxiety effect but this effect was not seen at higher doses, which was thought to be related to saffron sedative effects. Saffron (560 mg/kg) and safranal (0.05, 0.15 and 0.35 ml/kg, in a dose dependent manner) significantly potentiated pentobarbital-induced sleep. Saffron only decreased the motion balance and function in Rotarod method which indicates imbalance due to saffron muscle relaxing properties. In open field test, saffron decreased open field parameters, crocin reduced locomotion activities, grooming and leaning while safranal (0.05 and 0.15 ml/kg) reduced the activities but raised grooming, leaning and rearing behaviors. Overall, safranal showed hypnotic effects and it was concluded that safranal may bind to some benzodiazepines subtypes (BZ1, BZ2 and BZ3), however, it had no impact on muscle relaxation or motor imbalance (47).

*Antidepressant effects*

Hosseinzadeh *et al* (3) revealed that safranal owns significant antidepressant properties in a mouse model. In this research, an increase in swimming time in forced swimming test was thought to be related to amplified synaptic serotonin as it happens with fluoxetine. Also, an increase in climbing time was assumed to be due to synaptic noreadrenaline augmentation. In addition to these effects, more stereotypic activities were observed which are assumed to be a result of dopamine reuptake inhibition as bupropion acts partially via this pathway.

*Anticonvulsant effects*

In a study by Hosseinzadeh and Talebzadeh (48), administration of safranal showed significant protective effects against pentylenetetrazole-induced seizures, reduced seizure period and mortality percentage and delayed the onset of tonic convulsions.

Anti-absence effects of safranal (72.75, 145.5 or 291 mg/kg) on pharmacologically induced seizure (using γ-utyrolactone, baclofen or of GABA_A_ receptor antagonists; pentylenetetrazole, picrotoxin and bicuculline at low doses) were evaluated in C57BL/6 mice. To this end, the latency in seizure onset, spike and wave discharges duration and its interaction with GABAergic system were monitored. It was observed that safranal has no effect on electrocorticographic recording baseline. On the other hand, a significant decrease in spike and wave discharges duration in a dose-dependent manner was observed, except for picrotoxin induced seizure (49).

Using pentylenetetrazol (PTZ)-induced model of seizure it showed that treatment with safranal (145.5 and 291 mg/kg, IP) 15 mins prior to PTZ injection, results in an increased protection percentage and prolonged latency period in minimal clonic and generalized tonic–clonic seizures. In order to discuss more mechanistically, authors administered flumazenil (5 nmol, Intracerebroventriculary) and naloxone (5.5 nmol, Intracerebroventriculary and 2 mg/kg, IP) 15 min prior to safranal (145.5 mg/kg) injection. Flumazenil increased death rates leading to this hypothesis that safranal exerts some of its anticonvulsant effects through GABA_A_-benzodiazepine receptor complex, but naloxone pretreatment shows that opioid receptors play a minor role in anticonvulsant effects of safranal. Since monoterpenoids such as terpineol and linalool show depressant effects on CNS *in vivo* (50) and that linalool competitively inhibits glutamate receptors, it was suggested that safranal may demonstrate its impact on CNS via these mechanisms (51).

*Safranal binding sites in CNS*

**Figure 2:**
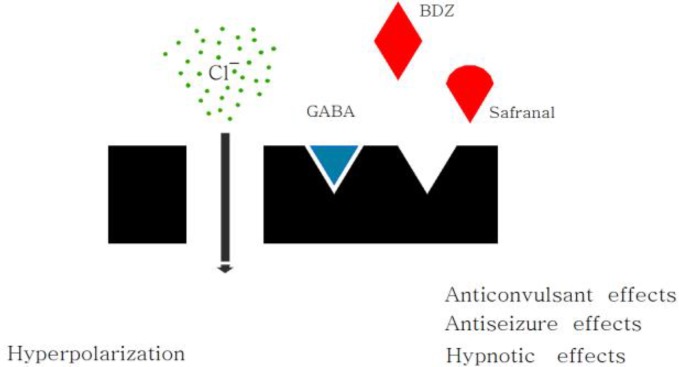
Safranal exhibits its anticonvulsant and hypnotic effects through binding the BDZ site of GABAA receptor

It has to be mentioned that while a single dose of safranal (291 mg/kg) reduced [^3^H] flunitrazepam binding in cortex, hippocampus and thalamus of mouse brain significantly, it did not change [^3^H] CGP 54626A binding. Authors suggested that safranal attenuates absence seizure via BDZ binding site of GABA_A _receptor complex (Figure 2), resulting in a recurrent inhibition in thalamic reticular nucleus and lessens its GABA_B_-mediated inhibitory effect onto the ventrobasal nuclei of the thalamus. Since safranal diminished [^3^H] flunitrazepam binding in three brain regions, it could be concluded that it boosts GABAergic inhibition in the cortex and/or hippocampus (49).

*Protective effect of safranal in CNS*

In a model of cerebral ischemia, safranal showed protective effects against oxidative damage,which was shown to be a result of an increase in sulfhydryl content and total antioxidant capacity and a decline in malondialdehyde (MDA) level in hippocampus. On the other hand, it was noticed that other monoterpenoids such as α-pinene have shown antioxidant activity and anti-inflammatory effects, as well (52).

*Safranal and morphine-withdrawal syndrome*

In order to evaluate the effects of safranal on morphine-withdrawal syndrome, Hosseinzadeh and Jahanian (8), made the mice morphine dependant by morphine administration at doses of 50, 50 and 75 mg/kg three times a day for three days and on the fourth day, 2 hr prior to the injection of naloxone, the last dose of morphine was administered. Safranal was administered at different doses 30 min before, 1 hr or 2 hr after the last dose of morphine. Based on results, safranal had the same effects as naloxone. Regarding the symptoms observed following safranal administration, the authors proposed that these serotonin-syndrome-like effects may be due to the co-administration of serotonergic antidepressants and opioids. Death was linked to potentiating morphine withdrawal syndrome and it was concluded that safranal acts as an opioid partial agonist.

*Safranal effects on memory*

A research by Hosseinzadeh and Ziaee (53) revealed that safranal (0.2 ml/kg) restored the impairing effect of scopolamine (1 mg/kg) on memory in the Morris water maze model in rats and showed no effect on intact memory, probably due to its sedative effect via BDZ site.

*Safranal and neurotransmitters release*

The extracellular concentrations of glutamate and aspartate (Excitatory Amino Acids, EAA) in hippocampus were assessed. In this study, rats received safranal (72.75 and 291 mg/kg, IP) and after 40 min KA (15 mg/kg, IP) was administered and EAA levels were recorded. Safranal did not affect the basal levels of EAA, but 80 min after administration of KA a significant rise in EAA levels was recorded which was significantly reduced by safranal (291 mg/kg, IP) (54). 

***Dermatologic effects***

Regarding the sunscreen and moisturizing properties of saffron, *C. sativus* pollens were dried, powdered and the SPF (sun protection factor) of saffron (2, 4 and 8%) was compared with homosalate (8%) lotion reference, using spectrophotometry method. Also moisture content of skin was evaluated at different times following topical administration of saffron. It was shown that saffron 4% has an equal SPF to homosalate 8% but saffron 8% has a significantly greater one than homosalate 8%. Skin moisture did not differ significantly after saffron topical administration (12). 

Based on the evidence that saffron is able to absorb the UV rays, Golmohammadzadeh *et al* (55), showed that there was no significant difference between SPF values of nanoliposomes containing 4% safranal and homosalate 8% standard sunscreen as measured by transpore tape method. Hence, it was concluded that safranal could be used as a natural sunscreen even at concentrations lower than of homosalate.

***Effects on sexual behavior***

Mounting frequency, intromission frequency, erection frequency, mount latency, intromission latency and ejaculation latency were evaluated following intraperitoneally administration of safranal (0.1, 0.2 and 0.4 ml/kg) to male rats. It was shown that safranal affects sexual behavior negatively and it was concluded that these effects might be because of safranal ability to inhibit serotonin reuptake, as delayed ejaculation, inability to ejaculate and absent or delayed orgasm is observed following administration of fluoxetine (9).

Another study by Shamsa *et al* (56) showed that taking saffron tablets (each tablet contains 200 mg dried saffron stigma) for ten days by twenty patients resulted in a statistically significant improvement in tip rigidity and tip tumescence as well as base rigidity and base tumescence. Authors proposed that this effect could be due to crocin and safranal antioxidant and radical scavenging properties and could be linked to their protective effect on ischemia-reperfusion injuries as it was observed in hind limb and kidney of rats.

**Table 4:** Summery of the effects of safranal on central nervous system

Effect (Reference)	Safranal dose	Results	Proposed mechanism
Anticonvulsant (51)	72.75, 145.5 and 291 mg/kg,	Decrease in the incidence of both minimal clonic seizures and generalized tonic–clonic seizures (GTCS). Full protection at higher doses against GTCS and death. Significant decrease in protective results against GTCS onset and rate when co-administered with flumazenil but not with naloxone.	Involvement of GABAA-benzodiazepine receptor complex
Anticonvulsant (49)	0.1, 0.2 and 0.4 ml/kg	No effect on electrocorticographic recording baseline. A significant decrease in spike and wave discharges duration in a dose-dependent manner, except for picrotoxin induced seizure	Binding to BDZ site of GABA complex, improving GABAergic inhibitory effects in cortex/hippocampus
Anticonvulsant (48)	0.15 and 0.35 ml/kg,	Reduction in duration of seizure and death rate. Increase in convulsion onset.	
Memory (53)	0.2 and 0.5 ml/kg	safranal (0.2 ml/kg) restored the impairing effect of scopolamine (1 mg/kg) on memory in the Morris water maze model in rats and showed no effect on intact memory	Safranal sedative effects
Antidepressant (3)	0.15, 0.35 and 0.5 ml/kg	Increase of immobility and swimming time, climbing time (0.5 ml/kg) and stereotypic activities	Amplification of synaptic serotonin, noreadrenaline Potentiation of dopaminergic system
Excitatory Amino Acids Release (54)	72.75 and 291 mg/kg	Reducing KA-induced raised levels of EAA	
Morphine-Withdrawal Syndrome (8)	0.0085-0.15 ml/kg	Induction of jumping, seizure, diarrhea, petosis, irritability, wet dog shake and death	Opioid partial agonism
Hypnotic (46)	90, 180 and 360 mg/kg	the duration of non-rapid eye movement (NREM) and the delta power activity of NREM sleep were increased and the NREM sleep latency was decreased. c-Fos expression was improved in the ventrolateral preoptic nucleus (VLPO) and decreased in the arousal histaminergic tuberomammillary nuclei (TMN).	potentiation of the sleep-promoting neurons in the VLPO alongside the inhibition of the wakefulness-promoting neurons in the TMN
Hypnotic/Antianxiety (47)	0.05, 0.15 and 0.35 ml/kg	Potentiating pentobarbital induced sleep, anti anxiety effects and reducing activities in open field.	Binding to benzodiazepines subtypes with no effect on muscle relaxation.
Cerebral Ischemia (52)	727.5 mg/kg, 363.75 mg/kg, 145.5 mg/kg, and 72.75 mg/kg,	Significant elevation of SH contents and antioxidant capacity and significant decrease of MDA level in hippocampus	Partially due to safranal potent depressant effect on CNS

***Effects on respiratory tract***

Safranal effects on respiratory tracts could be summarized as antitussive effects and H_1_ antagonism, which are shown in Table 5.

Safranal (0.2, 0.5 and 0.75 ml/kg) was administered to guinea pigs 30 min before the initiation of 10 min exposure to citric acid aerosol, as an irritant agent. Safranal significantly reduced the coughs count as compared to saline treated group (7).

Using pre-contracted isolated guinea pig tracheal chains, the β_2_-adrenergic stimulatory effects of safranal on isoprenaline-induced relaxation curves, was evaluated. Safranal (1.25 and 2.5 mg) significantly lowered isoprenaline EC_50_ and its maximum response as compared to saline. Since along with safranal, saffron aqueous-ethanolic extract was also tested in this study, this relatively potent stimulatory effect on β_2_-adrenoceptors was suggested to be due to possible inhibitory effect of *C. sativus* on histamine (H_1_) receptors (57).

The effect of safranal (0.63, 1.25 and 2.5 μg/ml) on histamine (H_1_) receptors was evaluated in guinea pig tracheal tissue in organ bath. Safranal resulted in rightward shifting in histamine–response curves, significant increase in maximum responses to histamine and greater EC_50_. It was concluded that safranal possibly acts as a histamine H_1_ receptors competitive antagonist (58). 

***Safranal antidotal effects***

In Wistar rats, treatment with acrylamide (50 mg/kg IP for 11days), significantly decreased body weight and induced severe gait abnormalities at the end of 11 days, but treatment with saffron extract (40, 80 and 160 mg/kg) reduced acrylamide-induced neurotoxicity in rats (59). 

Also, it has been shown that PC12 cells pretreatment with crocin (10, 20 and 50 µM) resulted in a significant protection against acrylamide cytotoxicity. In this study crocin inhibited ROS generation and increased Bax/Bcl2 ratio dose dependently (60). 

*Protection against diazinon toxicity*

To evaluate the possible protective effects of safranal against diazinon, safranal (0.025, 0.05 and 0.1 ml/kg) was administered IP to rats, three times a week, for four weeks. When co-administerd with diazinon, safranal 0.1 ml/kg significantly decreased AST, LDH, CPK and CPK-MB levels, safranal 0.025 ml/kg significantly decreased AST, ALT and GGT activities and safranal 0.05 ml/kg significantly decreased ALT when compared to the diazinon-treated group. At all doses, safranal significantly reduced the augmentation of direct 8-iso-prostaglandin F2α level caused by diazinon. Safranal made no change on the effect of diazinon on TNF-α level. Also pretreatment with safranal prevented the dizinon-induced S100β increase. Authors linked these results with the antioxidant properties of safranal, as it leads free radicals to attract a hydrogen atom from an antioxidant molecule rather than from polyunsaturated fatty acids and keeps them intact and so it is able to decrease the elevated levels of 8-iso-prostaglandin F2α which is a specific product of non-enzymatic lipid peroxidation (61).

With the same protocol of safranal administration, Hariri *et al* (62), observed that safranal 0.1 ml/kg alone or plus diazinon significantly reduced RBC, hemoglobin and hematocrit indices changed by diazinon while were reestablished by safranal 0.025 and 0.05 ml/kg significantly. Safranal 0.025 or 0.1 ml/kg plus diazinon increased platelets counts significantly when compared to diazinon treated group. At all doses safranal had no effects on diazinon impact on RBC cholinesterase activity. In the micronucleus assay, safranal could not reverse the genotoxicity of diazinon. Authors postulated that protective properties of safranal could be attributed to its radical scavenging effects on RBC count and hemoglobin concentration and also on bone marrow in production of platelets.

**Table 5:** Summary of the effects of safranal on respiratory tract

Effect (reference)	Safranal dose	Results	Proposed mechanism
Relaxant effect (58)	0.63, 1.25 and 2.5 μg/ml	Rightward shift in histamine–response curves	Antagonistim on histamine (H_1_) receptors
Relaxant effect (57)	1.25 and 2.5 mg	Increase in maximum responses to histamine and greater EC_50_	Inhibitory effect on histamine (H_1_) receptors
Antitussive (7)	2, 0.5 and 0.75 ml/kg	Reduction in coughs number	

*Against gentamicin-induced nephrotoxicity*

Boroushaki and Sadeghnia (63) investigated the effect of safranal on gentamicin-induced nephrotoxicity in rats. Animals were divided in group1: received saline (1 ml/kg), group 2: gentamicin (80 mg/kg/day; IP) and group 3: safranal (0.5 ml/kg; ip) followed by gentamicin (80 mg/kg/day; ip), for six days. On day 7, cardiac blood samples were collected and nephrotoxicity markers were evaluated. While blood urea nitrogen and creatinine and urinary glucose and protein concentration were significantly higher in gentamicin only treated group in comparison with saline treated, difference between saline treated and safranal plus gentamicin treated groups was not statistically significant. Authors declared that although the exact mechanism could not be described but this effect might be due to safranal antioxidant properties.

*Against hexachlorobutadiene -induced nephrotoxicity*

Boroushaki *et al* (64) used hexachlorobutadiene (HCBD) to cause nephrotoxicity in rats and evaluated the protective effects of safranal using kidneys for histological and MDA analysis and blood urine samples (for assessment of urea, creatinine, glucose and protein concentrations). Rats received a single dose of chemicals as follows: group 1: corn oil (1ml/kg), group 2: HCBD (50mg/kg) or group 3, 4 and 5: safranal (0.5, 0.25 and 0.1 ml/kg) 1 hr prior to HCBD (50mg/kg), IP. Blood creatinine levels were not statistically different among groups but blood urea was significantly lower in safranal treated groups (0.5, 0.25 ml/kg). Also, safranal (0.5, 0.25 ml/kg) lowered urinary levels of glucose and protein, significantly.

Light microscopic examination of kidneys' sections showed a massive damage in straight portion of proximal tubules in groups 2 and 5. Since no changes were observed in MDA results among different groups, it was concluded that HCBD nephrotoxicity could not be a result of oxidative stress. Hence, safranal antioxidant properties do not play an important role here. Although precise mechanism remains known but authors believed that inhibition of organic anion transporter (OAT) system and interference with the metabolism of HCBD via affecting the glutathione- S-transferase and/or cysteine conjugate β-lyase activity could justify safranal activity.

***Safranal metabolic effects***

Kianbakht and Hajiaghaee (65) evaluated six weeks administration of safranal (0.25 and 0.5 ml/kg/day, orally) effects on a model of alloxan- diabetic rats. Finally, while it was shown that safranal reduces fasting blood glucose and HbA1c levels and improves the blood insulin levels significantly, there were no significant changes in the blood SGOT, SGPT and creatinine levels. 

***Antihypertensive effect***

Imenshahidi *et al* (11) evaluated the effects of intravenously administered safranal (0.25, 0.5 and 1 mg/kg) on normotensive and desoxycorticosterone acetate-induced hypertensive rats. Safaranal at all doses decreased mean arterial blood pressure (MABP) significantly in hypertensive rats and at two higher doses in normotensive rats. Heart rate was also reduced at all doses but not statistically significant. Since saffron stigma aqueous extract and crocin were evaluated along with safranal and based on the results, it was proposed that safranal is more important than crocin in hypotensive properties of saffron. Authors mentioned that the immediate reduction in blood pressure suggests that both heart function and blood vessels contractility is affected by safranal. Based on the study of Boskabady and Aslani (66) in which a potent relaxant effect of safranal on smooth muscles of guinea pigs was shown and also because of interaction of safranal with GABA_A_-benzodiazepine receptor complex (48) and the observations that benzodiazepines, in pre-anesthetic doses, decrease blood pressure via decreasing peripheral resistance or cardiac output (67) hypotensive effect of safranal could be due to its interaction with GABA(A)-benzodiazepine receptor. Desoxycorticosterone acetate treatment causes an endothelial dysfunction both in the isolated aortic rings and perfused mesenteric bed (68) since safranal effects on normotensive and hypertensive rats were almost the same, it was concluded that its action is not endothelium dependent.

***Anti-ischemia***

Safranal (0.1–0.5 mL/kg) was administered to rats IP once daily for 14 days. On day 15, 45 min of ischemia was induced by one-stage ligation of left anterior descending coronary artery and afterward, reperfusion was carried out for 60 min. According to results, safranal reduced infarct size, enhanced left ventricular functions and myocardium hemodynamic status, generally. It was seen that following ischemia–reperfusion in myocardium, safranal increases phosphorylation of Akt/GSK-3b/eNOS and decreases IKK-b/NFқB protein expressions. Since safranal resulted in Bcl-2 expression upregulation and Bax and caspase3 expression downregulation, it could act as a strong antiapoptotic chemical. Also, safranal regularizes myocardial antioxidant and nitrotyrosine levels, cardiac injury markers (LDH and CK-MB), and decreased TNF-α level in a dose-dependent trend (69).

In a model of ischemia-reperfusion injury, safranal (0.1 ml/kg, 0.25 and 0.5 ml/kg, IP) administration before the injury (warm ischemia for 60 min followed by reperfusion for 90 min) significantly reduced thiobarbituric acid reactive species (as the marker of lipid peroxidation) which was confirmed by histopathological studies in which injury improvement was recorded (70).

In another study, hind limb ischemia was induced by clamping the common femoral artery and vein and was maintained for 2 hr. Following this period, rats experienced reperfusion for 1 hr. Safranal (0.1, 0.25 and 0.5 ml/kg) was administered 1 hr prior to repefusion. Safranal reduced MDA levels, increased the average peak-to-peak amplitudes of electromyographic (EMG) potentials and improved total sulfhydryl groups and antioxidant capacity of muscle (assessed by FRAP assay) significantly. Authors declared that safranal antioxidant effects could be attributed to its ability in quenching free radicals (71).

***Anti-nociceptive and anti-inflammatory effects***

It has been shown that both ethanolic and aqueous extracts of *C. sativus* petals and stigmas posses anti-nociceptive effects in acetic acid induced writhing. Weak to moderate anti inflammatory effects were observed using stigma extracts. Also, aqueous and ethanolic stigma extracts and ethanolic petal extract were found to be effective against chronic inflammation (72).

According to Nasri *et al* safranal exhibits anti-nociceptive effects in acute phase of formalin assay but has no effects on inflammation as assessed by Mercury immersion method (73).

Amin and Hosseinzadeh (74) evaluated the effects of systemic administration of safranal (0.025, 0.05 and 0.1 mg/kg) for 7 days in a rat model of neuropathic pain caused by chronic constriction injury. According to results, safranal attenuates the neuropathic pain behavioral symptoms, dose-dependently. It was assumed that modulation of BDZ sites on GABA_A_ receptors, decrease in aspartate and glutamate in hippocampus extracellular space which have been shown to be involved in the generation of neuropathic pain and antioxidant capacity of safranal may play an important role. 

Regarding antinociceptive effects of safranal, Hosseinzadeh and Shariaty (75) designed a study using hot-plate, writhing and formalin tests. Administration of safranal (0.1, 0.3 and 0.5 ml/kg/ip) to mice inhibited acetic acid-induced abdominal constrictions. Only 30 min after safranal 0.5 ml/kg treatment, pain threshold against the thermal source increased. In formalin test, pain-related behaviors were decreased in phase I by safranal 0.05 ml/kg/ and in phase II by safranal 0.05 and 0.025 ml/kg, significantly. Naloxone did not abolish the anti-nociceptive effects of safranal completely. It was concluded that safranal antinociceptive effects could be more due to its impact on prostaglandin synthesis or actions rather than opioid receptors. Based on formalin tests result, safranal has a greater impact on inflammatory and peripheral phase.

***Antimicrobial effect***

 In a research concerning saffron antimicrobial properties, Pintado *et al *(76) achieved safranal bactricidal effects at concentrations ranging between 8 mg/ml to 32 mg/ml on microorganisms namely *E. coli*, *S. aureus, S. aureus* 6538P, *S. enterica* serovars (Hadar FT33, Infantis, Typhimurium DT104, Typhimurium strain LT2, Virchow FT8).

***Antioxidant effects***

Assimopoulou *et al* (41) showed that safranal (500 ppm in methanol solution) possesses a 34% radical scavenging activity possibly due to its potentials to provide a hydrogen atom for the DPPH radical.

By means of deoxyribose, erythrocyte membrane lipid peroxidation and liver microsomal non-enzymatic lipid peroxide tion methods, the antioxidant activity of safranal (0.1, 0.5, 1 and 2 mM) was evaluated, *in vitro*. Safranal showed hydroxyl radical scavenging activity dose-dependently in deoxyribose assay and decreased MDA generation in RBC, lipid peroxidation induced by H2O2 and liver microsomal non-enzymatic lipid peroxidation (5).

***Safranal-DNA interactions***

The interaction between calf-thymus DNA and safranal (0.13 and 3.125 mM) under physiological conditions were evaluated by FTIR and UV-visible difference spectroscopic methods in order to assess binding sites and binding constants, also safranal effect on DNA duplex stability and conformation was monitored. Higher levels of DNA vibrations at higher safranal concentrations were described to be related to helix destabilization. According to results safranal-DNA binding constant was Ksafranal = 1.24 × 103 M-1 and it was mentioned that adducts stability for safranal was lower than that of crocetin. Despite the low calculated K values, following safranal treatment remarkable changes in DNA was observed (77).

Also another study was designed to evaluate safranal and picrocrocin interaction with calf thymus DNA (ctDNA) by means of Circular dichroism (CD) and fluorometric methods. Finally, nonintercalative/minor groove binding of these chemicals to ctDNA was observed. According to CD recordings, picrocrocin-ctDNA interaction takes place at lower concentrations than those of safranal. Regarding picrocrocin interaction with DNA/oligonucleotides, at higher concentrations B- to C-DNA transition and at lower concentrations unstacking of DNA and oligonucleotides bases were detected. Safranal- ctDNA interaction findings show (56% AT) and oligo(AT)15 interaction but for oligo(GC)15 , triple-helix DNA and B- to H-DNA transition were mentioned (78).

***Genoprotective effects***

Genoprotective effects of pretreatment with *C. sativus* stigma extract (CSSE) (5, 20, and 80 mg/Kg, ip) and crocin (50, 200, and 400 mg/Kg, IP) in comet assay (using methyl methanesulfonate (MMS) as a DNA damaging agent) has been shown by Hosseinzadeh *et al* (79). In this project, CSSE and crocin significantly reduced % tail DNA in a dose-dependent way which indicates a genoprotective effect. 

Hosseinzadeh and Sadeghnia (80) studied the genotoxic effects of safranal using single-cell gel electrophoresis (SCGE), comet assay. Male rats received safranal 72.75 mg/kg, IP alone or at the doses of 72.75 mg/kg and 363.75 mg/kg, IP 45 min prior to MMS (120 mg/kg, IP) administration. 3 hr after drug treatment, spleen, kidney, lung and liver were removed and nucleus suspension were prepared for comet assay in which DNA migration was measured. At all doses and in all organs cells, safranal pre-treatment resulted in a significant protection against MMS-induced DNA damage. It is worth mentioning that even at relatively high doses of safranal no adverse effect was recorded. 

***Toxicology***

According to Hosseinzadeh *et al* (81), subacute (21 days) and acute (2 days) toxicity of safranal were assessed using rat and mouse models. LD50 of safranal were calculated as follows:

Also, subacute toxicity of safranal were evaluated following once-daily administration of safranal (0.1, 0.25 or 0.5 ml/kg/day, orally) for 21 days. 5-7 min post treatment, rats expressed excitement and hyperactivity followed by sedation, relaxation and reduced locomotor activity. Also, asthenia, anorexia, decrease in food and water consumption and weight loss were observed. These effects were more pronounced at higher doses. Based on our results, there were a significant reduction in body weights, RBC, hemoglobin, hematocrit, and platelets at all doses. Differences in serum glucose, total bilirubin, serum creatinine, albumin, aspartate aminotransferase (AST), alanine aminotransferase (ALT), creatine phosphokinase (CPK), total bilirubin, MCV, MCH, MCHC and WBC between groups were not statistically significant. Safranal reduced total cholesterol, triglyceride at all doses and alkalin phosphatase (ALP) at two higher doses.

Also, safranal (0.5 ml/Kg) caused an increase in the levels of lactic acid dehydrogenase (LDH) and serum urea nitrogen (BUN) (81).

Regarding pathological assessment of heart, liver and spleen, no abnormalities were observed, but histological evaluations showed abnormalities and toxic effects of safranal, especially at the dose of 0.5 ml/Kg in kidneys and lung. In kidneys edema and cytolysis were recorded and in lungs progressed emphysema and lymphocyte infiltration were observed (81). 

Kanakis *et al* evaluated the safranal- human serum albumin interaction and their results showed that safranal binds nonspecifically (H-bonding) via protein polar groups with Ksaf = 2.11 (±0.35) × 10^3^ M^-1^ binding constant. At lower ligand concentration (1 μM) no change were made to protein secondary structure but some modifications were observed at higher concentration (1 mM). Also, The IC_50_ value of safranal was reported to be 95 ± 1 μg/mL (82). 

As for the genotoxicity, Hariri *et al* (62) showed that IP administration of safranal 0.1 ml/kg/day, three days per week for 4 weeks, results in an increase in micronucleus index.

*Cytotoxic effects*

Investigating cytotoxic and antimicrobial effects of safranal (0.075, 0.150, 0.300, 0.450, 0.600 and 0.800 mg/ml) Behravan *et al* (83) used brine shrimp assay (LD_50_ found to be 14.3±0.4 ppm) and calculated safranal MIC against *Agrobactrium tumefaciens *using microplate method (MIC= 1 mg/ml). In the potato disc assay, inhibitory effects of safranal on *Agrobactrium tumefaciens *tumors was evaluated (EC_50_= 0.31±0.04 mg/ml). Eventually it was concluded that safranal shows great dose-dependent anti-tumor and toxic effects of 90.2% anti-tumor effect at concentration of 0.800 mg/ml.

Another study was undertaken by Abdullaev *et al* (84) to evaluate saffron and its main constituent crocin and safranal antigenotoxic and cytotoxic properties, using the TA98 strain in the Ames/Salmonella test system, in the presence of 2-amino-antracene (2-AA) as a well known mutagen. The examination revealed that saffron extract 1500 mg/plate increased 2-AA’s mutagenic activity for more than 3 folds. Further investigations showed that safranal could be introduced as the chemical which plays the main role in this trend but no more explanations were provided.

**Table 6:** Safranal LD_50 _values

Root of administration	Male mice	Female mice	Male rats
Intraperitoneal	1.48 ml/kg	1.88 ml/kg	1.50 ml/kg
Oral	21.42 ml/kg	11.42 ml/kg	5.53 ml/kg

Escribano *et al* studied the cytotoxic effects of saffron ethanolic extract, crocin, safranal and picrocrocin at the concentration of LD_50_ (2.3 mg/ml, 3 mM, 0.8 mM and 3 mM, respectively) on Hela cells. Finally, it was concluded that crocin may have a more profound value as an anticancer agent in comparison with other main saffron chemicals (45).

## Conclusions

According to different studies, safranal as the main chemical responsible for *C.sativus* odor, exhibits different pharmacological activities i.e. anticonvulsant effects, hypnotic effects, etc. which justify its importance as a drug of future. As discussed above, the majority of studies is concerned with its effect on CNS and is based on the achievements in this field; safranal may be introduced as an anticonvulsant/anti anxiety/ hypnotic drug. To the best of our knowledge, missing areas of interest which have not been fully completed are clinical trials and toxicological studies, which need further attention.
